# Sagittal spinal-pelvic alignment in patients with Crowe type IV developmental dysplasia of the hip

**DOI:** 10.1186/s12891-020-03717-0

**Published:** 2020-10-17

**Authors:** Peng Ren, Xiangpeng Kong, Wei Chai, Yan Wang

**Affiliations:** 1 Medical School of Chinese PLA, 28 Fuxing Road, Beijing, 100853 China; 2 Department of Orthopaedics, the First Medical Center, Chinese PLA General Hospital, 28 Fuxing Road, Beijing, 100853 China

**Keywords:** Spinal-pelvic alignment, Sacral slope, Lumbar lordosis, Developmental dysplasia of the hip, Hip-spine syndrome

## Abstract

**Background:**

The impact of high dislocated dysplastic hips on spinal-pelvic alignment has not been well described. This study aims to evaluate compensatory spinal radiographic changes and presence of back pain in patients with Crowe type IV developmental dysplasia of the hip (DDH).

**Methods:**

An observational study was conducted from July 2016 to December 2017, and 49 consecutive patients with Crowe IV DDH were enrolled. Forty-nine sex- and age-matched asymptomatic healthy adults were recruited as the controls. The sacral slope (SS), lumbar lordosis (LL), spino-sacral angle (SSA), C7 tilt (C7T), and sagittal vertical axis (SVA [C7]) were measured on lateral whole spine radiographs. The presence of low back pain and visual analogue scale (VAS) scores were recorded.

**Results:**

The patients with Crowe IV DDH showed significantly greater SS (47.5 ± 7.5° vs. 40.4 ± 6.7°, *p* < 0.05), LL (− 63.7 ± 9.2° vs. − 53.3 ± 11.5°, *P* < 0.05), SSA (141.8° ± 7.2° vs. 130.6 ± 7.9°, *p* < 0.05), C7T (93.9 ± 3.6° vs. 91.1 ± 3.7°, *P* < 0.05), and lower SVA(C7) (− 16 mm[− 95–45] vs. 6.4 mm[− 52–47], *p* < 0.05) compared to the controls. The patients with bilateral Crowe IV DDH also exhibited larger SS, LL, SSA, and C7T and a smaller SVA (C7) than those with unilateral Crowe IV DDH. Sixty-three percent of the patients with Crowe IV DDH reported low back pain.

**Conclusion:**

The patients with Crowe IV DDH exhibited abnormal spinal-pelvic alignment characterized by anterior pelvic tilt, lumbar hyperlordosis, and a backward-leaning trunk. Bilateral Crowe IV DDH had a greater impact on spinal-pelvic alignment than unilateral Crowe IV DDH.

## Background

The compensatory mechanisms of the spine, pelvis, and lower limbs are essential in daily activities to maintain a stable, upright posture in the sagittal plane [1]. Sagittal spinal-pelvic alignment, first described in 1998, has been well studied in patients with spinal disorders, including low back pain, spondylolisthesis, and spinal deformities [2–5]. Patients with severe hip osteoarthritis (OA) have also been reported to have abnormal sagittal spinal-pelvic alignment [6, 7].

The incidence of hip dysplastic dislocation was reported to be between 0.1–0.15% in newborns and often involved unilateral side [8]. Girls are more likely to be involved than boys [9, 10]. In adults, high dislocated dysplastic hips are classified as Crowe IV developmental dysplasia of the hip (DDH), and may lead to hip pain, limp or wobbling gait [11]. Matsuyama reported changes in the sagittal alignment of the spine in patients with bilateral Crowe IV DDH [12]. The author observed that total sagittal alignment of the spine in these patients was compensated for by anterior tilt of the pelvis and lumbar hyperlordosis. In this regard, the main clinical symptom was lower back pain instead of leg pain [12]. To our knowledge, this is the only study that has focused on sagittal spinal alignment in these particular cases. However, the parameter representing pelvic tilt in his study was sacrum inclination (SI) angle, which was seldom reported in current literature. In addition, the patients with unilateral Crowe IV DDH were not included.

Thus, the observational study was conducted to evaluate compensatory spinal radiographic changes and presence of back pain in patients with Crowe IV DDH.

## Methods

This observational study was conducted from July 2016 to December 2017 with approval by the ethics committee at our institution, and informed consent was obtained from all subjects. Patients suffering from Crowe IV DDH who underwent total hip arthroplasty in our hospital were recruited. The exclusion criteria included the following: (1) a prior history of spinal surgery; (2) marked osteoarthritis in the knee or ankle; (3) lower limb radicular pain; (4) symptomatic spinal stenosis; (5) neurological disorders affecting postural control; (6) other diseases that would affect spinal-pelvic alignment, including lumbar disc herniation, spondylolisthesis, primary spinal deformity, severe lumbar spine degenerative change (Weiner Grade 3, which was performed by a senior surgeon reviewing the images and grading them according to the Weiner system.) [13], and a history of thoracolumbar fracture; and (7) patient preference by declining to participate in the study. During the study period, 52 patients were assessed and 49 patients who met the criteria were recruited consecutively into the study. Three patients were excluded, including one due to thoracolumbar kyphosis of 26°, one with a rigid scoliosis of 35° that involved the entire thoracic and lumbar spines, and one with L4/L5 and L5/S1 lumbar disc herniation. Based on the hip involved, patients were divided into two groups: patients with unilateral Crowe IV DDH (*n* = 39), and patients with bilateral Crowe IV DDH (*n* = 10).

Once enrolled, patients were asked about the presence and duration of chronic low back pain (LBP). Chronic LBP was defined as consecutive pain for greater than 3 months in the lumbar spine. The visual analogue scale (VAS) was used to assess pain intensity. Other frequently-used methods, such as the Oswestry Disability Index (ODI) and Roland-Morris Disability Questionnaire (RMDQ), were not utilized due to hip’s confounding effect. Forty-nine age (less than 2 years) and gender (one-to-one correspondence) matched healthy volunteers who fulfilled the following criteria were recruited as controls: (1) no pathology or surgical history of the pelvis, hip, or lower limbs; (2) no spinal trauma, deformity, or surgical history; (3) no spinal neurologic symptoms; (4) no severe lumbar spine degenerative change (Weiner Grade 3).

### Radiography and measurements

Standing lateral and anteroposterior full spine radiographs and oblique lumbar radiographs were obtained [14]. For the lateral view, patients stood in a natural, erect posture with knees in extension, and relaxed upper limbs with elbows half bent and hands resting on a support. For the anteroposterior view, patients stood in the same manner as the lateral view, with the exception being that the upper limbs were hanging alongside the body.

**Fig. 1:**
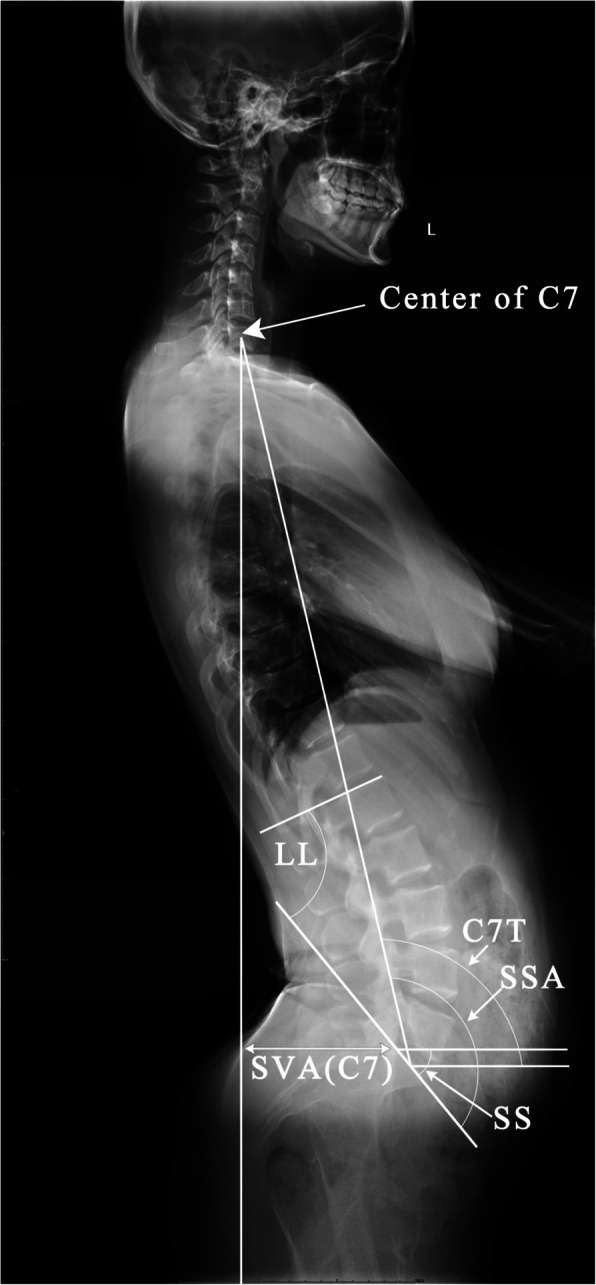
Illustration of the radiographic parameters of the spinal-pelvic alignment in bilateralhigh dislocated dysplastic hips. This patient has an anteriorly inclined pelvis, lumbar hyperlordosis, and a backward-leaning trunk. SS sacral slope, LL lumbar lordosis, C7T C7tilt, SSA spino-sacral angle, SVA(C7) sagittal vertical axis(C7)

**Fig. 2:**
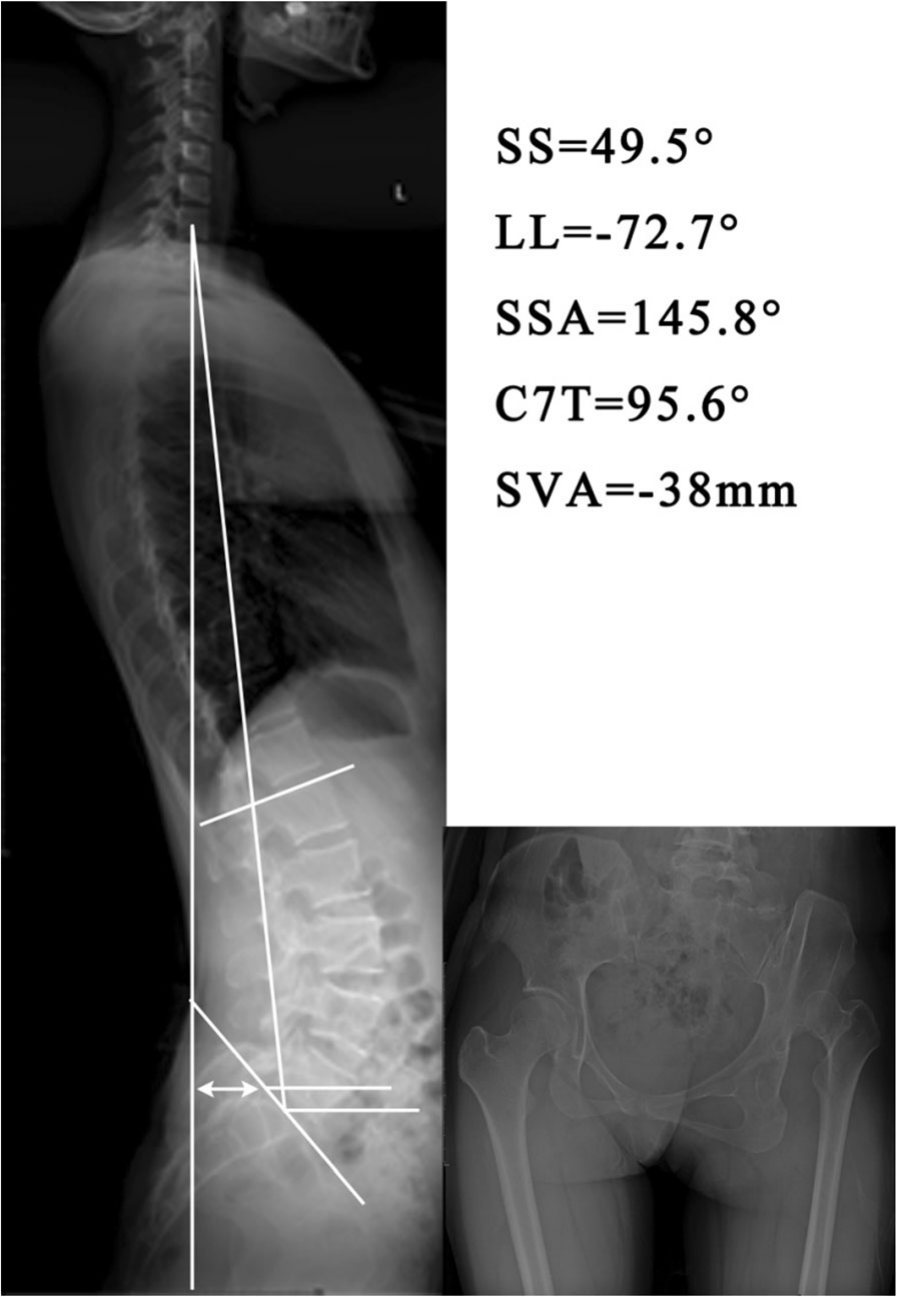
The sagittal spinal-pelvis alignment of a patient with unilateral hip high dislocated dysplasia. This patient had a significantly anterior tilted pelvis, lumbar hyperlordosis, and a backward-leaning trunk

The parameters of the sagittal spine-pelvis, including sacral slope (SS), lumbar lordosis (LL), spino-sacral angle (SSA), C7 tilt (C7T), and sagittal vertical axis (SVA [C7]) were measured by two independent observers on the lateral radiographs using the picture archiving and communication system (PACS) to assess inter-observer reliability. After 4 weeks, these measurements were repeated to assess intra-observer reliability. The methods used to measure these parameters are as follows (Figs. 1 and 2):

1. SS [2]: the angle between the sacral endplate and a horizontal line.
2. LL: the angle between the superior endplate of L1 and the S1 endplate.
3. SSA [15]: the angle between the sacral endplate and a line from the center of the C7 vertebral body to the center of the sacral endplate.
4. C7T [15]: the angle between the horizontal line and a line from the center of the C7 vertebral body to the center of the sacral endplate.
5. Sagittal vertical axis (SVA(C7)) [16]: the horizontal distance from the posterior edge of the sacral endplate to the plumb line passing through the center of the C7 vertebral body (C7PL).

Because pelvic incidence (PI) and pelvic tilt (PT) were the parameters relying on the normal relationship between femoral head and acetabulum, measuring PT and PI in Crowe IV DDH patients were inaccurate [2]. We didn’t include PT and PI in our study .

### Statistical analysis

All statistical analyses were performed by SPSS version 22 (IBM, Chicago, IL). Measurement data was expressed as the mean and standard deviation. Measurement data were analyzed by independent sample t test or analysis of variance. Categorical data was analyzed by the chi-square test. The intraclass correlation coeffificient (ICC) was used to determine the variations of the different measurements: 0.81 to 1.00 was regarded as nearly perfect reliability; 0.61 to 0.80, strong reliability; 0.41 to 0.60, moderate; 0.21 to 0.40, fair; and 0 to 0.20, poor. A *p*-value < 0.05 was considered significant.

## Results

General demographic data of the patients and controls are summarized in Table 1. There were 5 males and 44 females, and the average age was 39.9 ± 9.8 years (range 22–67 years). There were 39 patients (4 males and 35 females with an average age 40.1 ± 8.9 years [range 22–62 years]) with unilateral Crowe IV DDH, and 10 patients (10 females with an average age 39.2 ± 13.4 years [range 23–67 years]) with bilateral Crowe IV DDH. There were 5males and 44 females with an average age 39.9 ± 6.7 years (range 25–62 years) in the control group. The age and gender distributions between the groups revealed no significant differences.

**Table 1:** Patient demographics

	Unilateral Crowe IV DDH (*n* = 39)	Bilateral Crowe IV DDH (*n* = 10)	Control (*n* = 49)
Age			
Mean ± SD	40.1 ± 8.9	39.2 ± 13.3	39.9 ± 7.8
Range	22–62	23–67	20–57
Gender			
Male	4 (10.3%)	0 (0)	4 (8.2%)
Female	35 (90.7%)	10 (100%)	45 (91.8%)
BMI (kg/m^2^)	22.6 ± 4.0	21.2 ± 3.0	22.8 ± 3.2
Height	158.4 ± 6.9	157.4 ± 6.0	160 ± 6.3
weight	56 ± 11.2	53 ± 8.6	58 ± 10.7
Dislocated hip			
Left	18	10	–
Right	21	10	–
Non-dislocated hip			
Healthy	29	–	–
Crowe type I	2	–	–
Crowe type II	4	–	–
Crowe type III	4	–	–

The mean values and standard deviations of the radiographic parameters are showed in Table 2.

**Table 2:** Comparisons of the sagittal spinal–pelvic alignment parameters and LBP

	Control (*n* = 49)	Crow IV DDH		
		Total (*n* = 49)	Unilateral (*n* = 39)	Bilateral (*n* = 10)
SS(°)	40.4 ± 6.7	47.5 ± 7.5*	45.7 ± 7.2*	54.3 ± 2.8*^▲^
LL(°)	-53.3 ± 11.5	−63.7 ± 9.2*	−61.3 ± 8.8*	−72.9 ± 3.0*^▲^
SSA(°)	130.6 ± 7.9	141.8 ± 7.2*	139.7 ± 6.3*	150.2 ± 3.6*^▲^
C7T(°)	91.1 ± 3.7	93.9 ± 3.6*	93.3 ± 3.6*	96.3 ± 2.3*^▲^
SVA (mm)	6.4 (−52–47)	−16 (−95–45) *	−11.1(− 70–44.5) *	−32.6(−95–0) *^▲^
Number of patients with LBP		31 (63.2%)	24 (61.5%)	7 (70%)
Spine VAS scores of patients with LBP			5.3 ± 1.6	5.8 ± 1.7

Comparisons between Crow IV DDH patients and controls (independent sample t test). * *p* < 0.05

Comparisons between unilateral and bilateral Crowe IV DDH group. (independent sample t test).^▲^*p* < 0.05

There were no differences in prevalence (*p* = 0.13) or low back pain intensity based on VAS (*p* = 0.48) between patients with low back pain in unilateral and bilateral Crowe IV DDH group

*DDH* developmental dysplasia of the hip, *LBP* low back pain, *VAS* visual analogue scale

Compared to asymptomatic controls, the patients with Crowe IV DDH showed significantly greater SS, LL, SSA, C7T, and lower SVA(C7). Among Crowe IV DDH patients, the bilateral had a significantly greater SS, LL, SSA, and C7T and lower SVA(C7) than the unilateral.

Reliability analysis demonstrated that the ICC of both inter-observer agreements and intra-observer agreements were larger than 0.81.

Thirty-one (63.2%) patients with Crowe IV DDH reported LBP, including 24 patients with unilateral Crowe IV DDH and 7 with bilateral Crowe IV DDH. Among the patients with LBP, the unilateral and bilateral Crowe IV DDH patients had an average VAS of 5.3 ± 1.6 and 5.8 ± 1.7, respectively. There were no differences in prevalence or VAS between unilateral and bilateral Crowe IV DDH group.

## Discussion

In this study, we found that the patients with Crowe IV DDH showed significantly greater SS, LL, SSA, C7T, and lower SVA(C7) than controls, which was more obvious in the patients with bilateral Crowe IV DDH. Thirty-one (63.2%) Crowe IV DDH patients reported LBP. However, there were no significant differences in prevalence or VAS between unilateral and bilateral Crowe IV DDH group.

Several studies reported the impact of hip disease on spine-pelvic alignments, and their results were inconsistent. In a prospective study, Weng reported a significantly larger SS and smaller PT in patients with hip OA [6]. Piazzolla reported that patients with hip OA and low back pain have significantly increased SS, LL, and forward inclination of the trunk [17]. Yoshimoto chose individuals with low back pain as controls and reported that patients with hip OA showed significantly higher PI, SS and LL [7]. However, Sariali found that patients with hip OA had significantly lower SS than asymptomatic healthy controls [18]. In patients with secondary OA with hip dysplasia, Okuda found that pelvic inclination tended to increase in pre/early-stage OA patients. With aging, patients with OA maintained the lumbar lordotic angle and did not develop a posterior sacral slope angle [19]. Our results showed the patients with Crowe IV DDH have significantly increased SS, LL.

Crowe IV DDH may represent one of the most severe conditions among the spectrum of hip pathology. One previous study found that total sagittal alignment of the spine in patients with bilateral hip dislocations was compensated for by anterior tilt of the pelvis and lumbar hyperlordosis [12]. However, the parameter SI, which was used to evaluate sacral rotation, was not frequently reported in the recent literature.

The present study used SS to evaluate pelvic rotation. Our results revealed that the patients with Crowe IV DDH had significantly larger SS than that of controls. As a parameter closely correlated with SS, the LL angle showed the similar results. Our results also indicated that even with unilateral dislocated hip, the spinal-pelvic alignment can be altered. However, with one non-dislocated hip sustaining the trunk, the pelvic orientation in unilateral cases may not change as much as bilateral cases.

SVA(C7) was measured in the present study to assess the global spinal balance. The spine is considered slightly unbalanced if C7PL located between the femoral head and the posterior edge of the sacral plate, and severely unbalanced if it is located anterior to the femoral heads. Our results revealed the patients with Crowe IV DDH had an average lower SVA than control group. This means the patients with Crowe IV DDH present with a tendency toward a backward-leaning trunk, which was in accordance with the description of the relationship between femoral head, central gravity line, and C7PL [20].

Other parameters related to C7 were C7T and SSA. C7T is a functional parameter that reflects the global orientation of spine. SSA is a parameter that quantifies the global kyphosis of the entire spine and pelvis. C7T, SSA and SS are linked by the following equation: SSA = C7T + SS [16]. In the present study, the average SSA and C7T in the patients with Crowe IV DDH were significantly larger than those in the control group. This result also indicated that patients with Crowe IV DDH present with a more backward global orientation of the spine, and besides, a decreased global kyphosis of the entire spine-pelvis.

Hip diseases would lead to the change of sagittal spinal-pelvic alignment, while different diseases may have different mechanisms for this change. Hip flexion contracture has been reported as one cause of pelvic anteversion and lumbar hyperlordosis in patients with severe hip OA [21]. However, severe dysplastic hips often reveal a greater range of motion (ROM) than healthy hips. Therefore, spinal alignment changes in Crowe IV DDH cannot be attributed to hip flexion contracture. In the Crowe IV DDH patients, the femoral head is dislocated upward and backward, and the trunk supporting function of hip joint loses. Meanwhile, anatomical course of soft tissue also changed. It is the two factors that make the pelvic rotation anteriorly and thereby increase SS. Lumbar lordosis will then be increased to compensate for the anterior pelvic tilt and to meet the criterion of central gravity line passing approximately through the femoral head.

LBP caused by hip disease was termed “secondary” Hip-Spine Syndrome (HSS) by Offierski and MacNab [21]. They suggested that hyperlordosis of the lumbar spine may result in abnormal force on the posterior facets and LBP. Parvizi reported that 49% of patients with end-stage hip arthritis had LBP [22]. In the patients with Crowe IV DDH, the current authors considered that several factors may contribute to LBP, the first factor was the overloaded shear and compressing stresses on the posterior facet joints; the second factor was the asymmetric load distribution on posterior facet joints; the third factor was the fatigue of back muscles. Therefore, although the average age of the Crowe IV DDH patients in our study was only 40 years old, the observed LBP prevalence was 63.2%.

There are several limitations in the present study. Firstly, several parameters related to hip centers were not measured, including PI and PT. Measuring PT and PI in the Crowe IVDDH patients were inaccurate due to the high dislocated hip joints. Secondly, though ages in our groups varied quite a bit, and the status of the contralateral hip differed in patients with unilateral hip dislocation, we did not set subgroups because of the small sample size. Thirdly, we did not attempt to explore the correlation between lumbago and the spinal-pelvic alignment. LBP can be caused by multiple factors, which would be taken into account in future research.

## Conclusion

The patients with Crowe IV DDH exhibited abnormal spinal-pelvic alignment characterized by anterior pelvic tilt, lumbar hyperlordosis, and a backward-leaning trunk. Bilateral Crowe IV DDH had a greater impact on spinal-pelvic alignment than unilateral Crowe IV DDH.

## Data Availability

The datasets analysed during the current study are available from the corresponding author on reasonable request.

## Abbreviations

DDH: Developmental dysplasia of the hip
THA: Total hip arthroplasty
SS: sacral slope
LL: Lumbar lordosis
SSA: Spino-sacral angle
C7T: C7 tilt
SVA: Sagittal vertical axis
PT: Pelvic tilt
PI: Pelvic incidence
SI: Sacrum inclination
OA: Osteoarthritis
LBP: Low back pain
VAS: Visual analogue scale
ODI: Oswestry disability index
RMDQ: Roland-Morris disability questionnaire
C7PL: C7 plumbline
PACS: Picture archiving and communication system
ROM: Range of motion
HSS: Hip-Spine Syndrome
ICC: Intraclass correlation coeffificient
